# Effect Modification and Its Impact on Preventable and Attributable Fractions in the Potential Outcomes Framework

**DOI:** 10.2188/jea.JE20250409

**Published:** 2026-05-05

**Authors:** Bronner P. Gonçalves, Etsuji Suzuki

**Affiliations:** 1 Faculty of Health and Medical Sciences, University of Surrey, Guildford, United Kingdom; 2 Department of Epidemiology, Graduate School of Medicine, Dentistry and Pharmaceutical Sciences, Okayama University, Okayama, Japan

**Keywords:** preventable fraction, attributable fraction, effect modification, causality

## Abstract

**Background:**

Policy decisions should be guided by measures that capture the impact of exposures on outcomes and that explicitly account for present-day exposure distribution. Both the preventable and attributable fractions have been used for this purpose; however, exposure effects can vary across subpopulations, and when this occurs, appropriate interpretation of these measures should be facilitated by a discussion of the contributions of different subpopulations.

**Methods:**

We analyze preventable and attributable fractions in the presence of effect modification. In particular, we use potential outcomes to formally define these quantities and to clarify the weighting of different strata in the total population measures.

**Results:**

Our derivations show that stratum-specific preventable and attributable fractions are weighted in proportion to the relative frequencies of effect modifiers among individuals with the outcome of interest. We also demonstrate that these weights are valid for the related quantities, preventable and attributable proportions. Finally, we present an example that illustrates how effect modification affects interpretation of these measures.

**Conclusion:**

In sum, when effect modification is present, investigators should consider reporting these measures by the relevant population strata, and information that would allow quantification of their implicit weights in the total population estimate. Our study provides a formal justification for this approach.

## INTRODUCTION

Effect modification is relevant to much epidemiologic research that aims to make causal inferences,^1^^–^^4^ and the implied heterogeneity of effects in different population strata may influence interpretation and use of study findings. For example, public health professionals often need to carefully consider the benefits of introducing a new treatment whenever evidence suggests considerable variation in population stratum-specific average treatment effects. Further, questions on generalizability and transportability also require understanding effect modification.^5^^–^^9^ The importance of this notion, however, is not limited to traditional causal effect measures. Specifically, the study of estimands that do not correspond to contrasts of counterfactual quantities, but rather correspond to contrasts of factual versus counterfactual quantities (see Rudolph et al^10^ for a related discussion on natural course effects), would also benefit from explicit considerations on effect modification.

Here, we use the potential outcomes framework^11^ to investigate how two of these estimands, namely the preventable fraction (a measure that is relevant for preventive exposures or interventions) and the attributable fraction (a measure relevant for harmful exposures), are affected by the presence of effect modifiers.

## METHODS

In this section, we provide formal definitions of the preventable fraction and attributable fraction under the potential outcome framework.^12^^–^^14^ Below, formulas and derivations refer to a binary exposure denoted by *A* (1 = exposed, 0 = unexposed), and to a binary outcome, denoted by *Y* (1 = disease, 0 = absence of disease). Further, *Y^a^* represents the potential outcome had *A* been set to *a*. Throughout the article, we assume that consistency holds (ie, *Y^a^* = *Y* if *A* = *a* [*a* = 0,1]).

### Potential outcomes-based definitions

The policy-relevant epidemiologic measures of preventable fraction for the total population (*PF_total_*) and attributable fraction for the total population (*AF_total_*) have been used in epidemiologic research for decades^15^^–^^19^ but only recently were formalized using potential outcomes.^20^^–^^26^ These two measures can be viewed as examples of what has been referred to as “population intervention effects”^27^ and are generally used to examine the potential population-level impact of exposures. Concretely, *PF_total_* is defined as the number of disease cases that would be prevented had the entire population been given a treatment relative to the number of observed cases^20^^,^^28^ (derivation of all formulas is presented in eMaterial 1):

$$\begin{array}{rl} {PF}_{\text{total}} & ≜\frac{\mathrm{Pr}(Y=1)-\mathrm{Pr}(Y^{1}=1)}{\mathrm{Pr}(Y=1)}\\ & =\frac{\mathrm{Pr}(A=0)(\mathrm{Pr}(Y=1|A=0)-\mathrm{Pr}(Y^{1}=1|A=0))}{\mathrm{Pr}(Y=1)}. \end{array}$$

By using potential outcomes,^13^ one can readily see that the definition of *PF_total_* involves the contrast of a factual term (Pr(*Y* = 1)) versus a counterfactual term (Pr(*Y*^1^ = 1)). Moreover, in the second equality, the magnitude of *PF_total_* depends on the quantity Pr(*Y* = 1|*A* = 0) − Pr(*Y*^1^ = 1|*A* = 0), which, under the consistency assumption, is equivalent to the causal risk difference of the exposure on the outcome for those who are actually unexposed (ie, Pr(*Y*^0^ = 1|*A* = 0) − Pr(*Y*^1^ = 1|*A* = 0)).

The attributable fraction for the total population (*AF_total_*), in its turn, is defined as the relative reduction in the number of disease cases had the (harmful) exposure been absent. Formally,

$$\begin{array}{rl} {AF}_{\text{total}} & ≜\frac{\mathrm{Pr}(Y=1)-\mathrm{Pr}(Y^{0}=1)}{\mathrm{Pr}(Y=1)}\\ & =\frac{\mathrm{Pr}(A=1)(\mathrm{Pr}(Y=1|A=1)-\mathrm{Pr}(Y^{0}=1|A=1))}{\mathrm{Pr}(Y=1)}. \end{array}$$

As emphasized in the article by Suzuki and Yamamoto,^20^ when coding of the exposure is reversed, the preventable fraction becomes equivalent to the attributable fraction. Further, the targets of causation differ between these measures: the unexposed group in the preventable fraction, and the exposed group in the attributable fraction. In eFigure 1, we provide a graphical summary of the relations between these measures, average causal effects, target populations, and target groups of causation.

Finally, note that while we present these measures as contrasts between factual and counterfactual quantities, others might conceive of them as contrasts between two counterfactual quantities because, under the consistency assumption, Pr(*Y* = 1) can be written using potential outcomes as Pr(*A* = 1)Pr(*Y*^1^ = 1|*A* = 1) + Pr(*A* = 0)Pr(*Y*^0^ = 1|*A* = 0) (see also a discussion on the model-based approach for estimation of natural-course effects in Rudolph et al^10^). Our findings remain valid irrespective of these differing interpretations.

## RESULTS

### Preventable fraction

As mentioned above, effects of an exposure or treatment on a health outcome are not generally homogenous in a population and can vary by levels of factors associated with the outcome (effect modifiers; see VanderWeele and Robins^29^ for causal structures that can lead to effect modification). To express the preventable fraction in the total population, which is relevant when the exposure is protective in the unexposed group,^20^ in terms of parameters defined in strata of potential effect modifiers, firstly, note that from the law of total probability, the following holds:

$${PF}_{\text{total}}=\frac{\mathrm{Pr}(A=0)\sum_{m}\mathrm{Pr}(M=m|A=0)(\mathrm{Pr}(Y=1|A=0,M=m)-\mathrm{Pr}(Y^{1}=1|A=0,M=m))}{\mathrm{Pr}(Y=1)},$$ (Expression 1)

where *M* is a (set of) covariate(s). Since our focus here is on effect modification, we assume that *M* is an effect modifier in the risk difference measure^30^ for those who are actually unexposed in the following discussion, which means that Pr(*Y*^0^ = 1|*A* = 0, *M* = *m*) − Pr(*Y*^1^ = 1|*A* = 0, *M* = *m*) varies with levels of *M*. Under the consistency assumption, this quantity can be rewritten as Pr(*Y* = 1|*A* = 0, *M* = *m*) − Pr(*Y*^1^ = 1|*A* = 0, *M* = *m*), as shown in the numerator of Expression 1. Furthermore, note that even in settings where *M* is an effect modifier, other effect modifiers might exist that are not included in *M*. Now, let *PF_m_* represent the preventable fraction for individuals with *M* = *m*:

$${PF}_{m}≜\frac{\mathrm{Pr}(Y=1|M=m)-\mathrm{Pr}(Y^{1}=1|M=m)}{\mathrm{Pr}(Y=1|M=m)}=\frac{\mathrm{Pr}(A=0|M=m)(\mathrm{Pr}(Y=1|A=0,M=m)-\mathrm{Pr}(Y^{1}=1|A=0,M=m))}{\mathrm{Pr}(Y=1|M=m)}.$$

Consistent with previous discussions^20^^,^^31^ and analogous to a comment above on the *PF_total_*, while the target population of *PF_m_* is the subpopulation with *M* = *m*, the target of causation is the unexposed subpopulation with *M* = *m*. Using Bayes rule, then:

$${PF}_{m}=\frac{\mathrm{Pr}(A=0)\mathrm{Pr}(M=m|A=0)(\mathrm{Pr}(Y=1|A=0,M=m)-\mathrm{Pr}(Y^{1}=1|A=0,M=m))}{\mathrm{Pr}(Y=1,M=m)}.$$ (Expression 2)

Using Expression 1 and Expression 2, it can then be shown that *PF_total_* is a weighted average of *PF_m_*, with weights corresponding to *M* level frequencies among individuals with the outcome:

$$\begin{array}{rl} {PF}_{\text{total}} & =\sum_{m}\frac{{PF}_{m}\mathrm{Pr}(Y=1,M=m)}{\mathrm{Pr}(Y=1)}\\ & =\sum_{m}{PF}_{m}\mathrm{Pr}(M=m|Y=1). \end{array}$$ (Expression 3)

In words, the weighting does not directly depend on population-level frequencies of *M* (ie, Pr(*M* = *m*)), but rather on the proportion of cases with level *m* (although this latter quantity ultimately depends on the former). Note that in the article by Suzuki and Yamamoto,^20^ related formulas expressed in terms of standardized risk ratio in the unexposed group were presented.

### Attributable fraction

In eMaterial 1, we show that a similar result holds for the attributable fraction, which is relevant when the exposure *A* is harmful for the exposed group.^20^ In particular, we show that

$${AF}_{\text{total}}=\sum_{m}{AF}_{m}\mathrm{Pr}(M=m|Y=1),$$ (Expression 4)

where *AF_m_* denotes the attributable fraction for the subpopulation with level *m* of *M*.

### Preventable and attributable proportions

Note that the definitions of *PF_total_* and *AF_total_* above correspond to the preventable caseload (population) and attributable caseload (population), respectively.^21^ Indeed, as can be seen in the formulas defining *PF_total_* and *AF_total_*, the numerators are not contained in the denominators, and the fractions do not correspond to proportions. Two related measures with ranges from 0 to 1 are the preventable proportion for the total population (*PP_total_*)^23^ and the attributable proportion for the total population (*AP_total_*):

$${PP}_{\text{total}}≜\frac{\mathrm{Pr}(Y=1)-\mathrm{Pr}(Y=1,Y^{1}=1)}{\mathrm{Pr}(Y=1)},$$

$${AP}_{\text{total}}≜\frac{\mathrm{Pr}(Y=1)-\mathrm{Pr}(Y=1,Y^{0}=1)}{\mathrm{Pr}(Y=1)}.$$

In eMaterial 1, we show that, in the presence of effect modification, the weights derived above for caseloads are also valid for these measures.

### Numerical example

To illustrate implications of these results, in Table 1 we present a hypothetical example of a clinical trial to assess the effect of a preventive treatment or exposure *A* on outcome *Y*, where *A* was marginally randomized. We assume that study enrolment was dependent on a variable *M*, with three levels, where *M* is an effect modifier in the risk difference measure; individuals with *M* = 0 or *M* = 1, who were assumed to have worse prognosis, were prioritized for recruitment over individuals with *M* = 2. Because *A* is a preventive treatment or exposure, we focus on the preventable fraction here. Note that, by the weak union graphoid axiom,^32^ exchangeability holds within levels of the variable *M* as shown below:

$$\left(Y^{a},M)⊥\;\;\;⊥A\;(a=0,1)\Rightarrow Y^{a}⊥\;\;\;⊥A|M\;(a=0,1\right)$$

Although the proportions of individuals with *M* = 0, 1 and 2 (ie, Pr(*M* = *m*)) were respectively 0.50, 0.30, and 0.20, the weights (ie, Pr(*M* = *m*|*Y* = 1)) used for *PF_m_*_=0_ and *PF_m_*_=1_ were 0.66 and 0.25, respectively, and for *PF_m_*_=2_ the weight was only 0.09, reflecting the fact that the observed risk of the outcome was higher for individuals with *M* = 0. The impact of preferential recruitment of high-risk individuals on the preventable fraction in the total population is therefore magnified by the extent to which these individuals’ risk is higher. Moreover, note that, under the consistency assumption, the stratum-specific effect in the unexposed, Pr(*Y* = 1|*A* = 0, *M* = *m*) − Pr(*Y*^1^ = 1|*A* = 0, *M* = *m*), is strongest in individuals *M* = 0, and, as can be seen in Expression 2, this explains why the stratum-specific preventable fraction for participants with *M* = 0 is higher than the preventable fraction in the other two strata.

**Table 1.** Data from a hypothetical clinical trial

*M*	*A*	Pr(*A* = *a*, *M* = *m*)	Pr(*Y* = 1|*A* = *a*, *M* = *m*)	Pr(*Y*^1^ = 1|*A* = *a*, *M* = *m*)	Pr(*Y* = 1|*M* = *m*)	*PF_m_*	Pr(*M* = *m*|*Y* = 1)	*PF_total_*
0	0	0.25	0.50	0.30	0.40	0.25	0.66	
	1	0.25	0.30	0.30				

1	0	0.15	0.30	0.20	0.25	0.20	0.25	
	1	0.15	0.20	0.20				

2	0	0.10	0.16	0.12	0.14	0.14	0.09	
	1	0.10	0.12	0.12				

			Pr(*Y* = 1)	Pr(*Y*^1^ = 1)				
Total		1.00	0.30	0.23			1.00	0.23

Quantities necessary for the calculation of the preventable fraction in the total population and in the three strata defined by the variable *M* are shown in different columns; rounding to two decimal places was used for some numbers in the table. The probabilities presented in the third, fourth, fifth, sixth and eighth columns correspond, respectively, to: the joint probability of *A* = *a* and *M* = *m*; the observational probability that *Y* = 1 by strata defined by (*A* = *a*, *M* = *m*); the probability that the potential outcome *Y*^1^ equals 1 in strata defined by (*A* = *a*, *M* = *m*); the observational probability that *Y* = 1 by strata defined by *M* = *m*; and the proportion of levels of *M* among individuals observed to have the outcome. In the hypothetical trial, *A* was marginally randomized, and individuals had an equal probability of being assigned to each level of *A* (ie, Pr(*A* = *a*) = Pr(*A* = *a*|*M* = *m*) = 0.5). Note that the *PF_total_* shown in the rightmost column, which was calculated using Expression 3, could also be obtained from quantities in the bottom row, Pr(*Y* = 1) and Pr(*Y*^1^ = 1).

## DISCUSSION

This example illustrates two consequences of the presence of effect modification for the interpretation of the preventable (and attributable) fraction that are important for policy decision making. First, even in situations where proportions of exposure do not vary in the different strata (ie, Pr(*A* = *a*) = Pr(*A* = *a*|*M* = *m*)), subpopulation-specific preventable fractions are likely to differ among the various groups; this follows directly from the modification of the corresponding effect by the factor used for stratification. In particular, in our numerical example, the stratum with the strongest preventive exposure effect also had the highest *PF_m_*, despite having the highest value for the denominator of *PF_m_*. Second, when effect heterogeneity is present and relevant, the quantity that is usually reported in epidemiologic studies, the preventable fraction for the total population (or the attributable fraction for the total population), can be understood as a weighted average of stratum-specific estimates, with the groups of unexposed (exposed, if interest lies in the attributable fraction) individuals at higher risk of disease disproportionally contributing to the total population estimate. For instance, in the numerical example above, while the preventable fraction for the entire population is 0.23, reporting of results also by strata of *M* would allow identification of a stratum of the population, with 20% of individuals, for which the preventable fraction is considerably lower, and this could have implications for resource allocation.

Such considerations are all the more important given the centrality of preventive exposures, such as vaccines, in public health emergencies. For instance, it has been suggested that vaccine efficacy against disease caused by the severe acute respiratory syndrome coronavirus 2 (SARS-CoV-2) might be lower for individuals with prior SARS-CoV-2 infections compared to individuals with no previous exposure to this virus.^33^ The derivation presented above implies that in future studies that will quantify the preventable fraction due to vaccines for coronavirus disease 2019 outcomes, investigators should also report estimates by strata defined by history of SARS-CoV-2 infections, so that readers and policymakers can better understand the preventable fraction for the total population.

More generally, our work advances the literature on these important epidemiologic measures by using one of the main frameworks for causal inference, the potential outcomes framework, to formally derive expressions that provide insights into how different population strata contribute to the measures that apply to the total population. Here, it is important to mention that although formulas have been reported that are similar to those in this paper,^16^ previous studies did not use causal formalism in their derivations and generally focused on attributable fractions, rather than on preventable fractions or the related proportion-based measures (*PP_total_* and *AP_total_*). For example, in a review by Benichou,^17^ one of the stratification approaches involved weights similar to those derived above; however, potential outcomes were not discussed in that study. Moreover, the study that first described the approach also did not use potential outcomes in the derivation of the weights.^18^ Further, note the apparent similarity of the weights derived here to those reported in the paper by Rockhill and colleagues^19^ that refer to attributable fractions for multicategory exposures; nevertheless, the interpretation is different, as the quantities being weighted represent attributable fractions for individuals with particular levels of exposure. Additionally, although our focus is on effect modification and we do not consider the presence of confounding, Darrow and Steenland^34^ discussed the impact of confounding on the attributable fraction assuming no effect modification and did not use potential outcomes. Finally, even though formulas reported here are general and do not presuppose particular estimation methods for these measures, it is worth noting that partial exchangeability under no exposure (ie, $Y^{0}⊥\;\;\;⊥A$ ) is a necessary and sufficient condition for both the Miettinen formula and Levin formula to yield the attributable fraction^20^ (see also a related discussion on errors in the calculation of attributable fractions^31^^,^^35^^–^^37^). Analogous discussion for the preventable fraction was also provided in Suzuki and Yamamoto.^20^

To conclude, the fact that these measures have been analyzed for several decades is a testament to their usefulness in public health practice,^15^ and our hope is that this work will facilitate their interpretation in contexts where effect modification is present and might be important for planning deployment of interventions.

## SUPPLEMENTARY MATERIAL

eMaterial 1. Derivation methods

eFigure 1. Relationships between attributable and preventable fractions, average causal effects (ACE), target population, and target group of causation. Regarding target populations, we assume that both *AF_total_* and *PF_total_* as well as the average causal effects under consideration are defined for the entire population; that is, they are not defined via conditioning on other variables, including potential outcomes variables. By target of causation, it is meant the subset of the population for which there must be a non-zero contrast between the (average) observed and the relevant counterfactual outcomes, so that the *AF_total_*, or *PF_total_*, is non-zero; for the average causal effect, this corresponds to (the potential subset of) the population included in the contrast of average potential outcomes.
