# Supplementary material for: Effect Modification and Its Impact on Preventable and Attributable Fractions in the Potential Outcomes Framework
Source: J Epidemiol. 2026 May 5;36(5):168–72. doi: 10.2188/jea.JE20250409 (PMC13085676; doi:10.2188/jea.JE20250409)

eMaterial 1. Derivation methods

Derivation for the preventable fraction

First, under the consistency assumption, the preventable fraction in the total population can be expressed in terms of the strata of M as follows:

$$
\begin{aligned}
PF_{total} &\triangleq \frac{\Pr(Y=1)-\Pr(Y^1=1)}{\Pr(Y=1)} \\
&= \frac{\sum_{a=0}^{1}\Pr(Y=1\mid A=a)\Pr(A=a) - \sum_{a=0}^{1}\Pr(Y^1=1\mid A=a)\Pr(A=a)}{\Pr(Y=1)} \\
&= \frac{\Pr(A=0)\,(\Pr(Y=1\mid A=0) - \Pr(Y^1=1\mid A=0))}{\Pr(Y=1)} \\
&= \frac{\Pr(A=0)\sum_m(\Pr(Y=1\mid A=0,\ M=m)-\Pr(Y^1=1\mid A=0,\ M=m))\Pr(M=m|A=0)}{\Pr(Y=1)}.
\end{aligned}
$$

The third equation holds because $\Pr(Y=1\mid A=1)=\Pr(Y^1=1\mid A=1)$ holds. Similarly, the m-level preventable fraction is expressed as follows:

$$
\begin{aligned}
PF_m &\triangleq \frac{\Pr(Y=1|M=m)-\Pr(Y^1=1|M=m)}{\Pr(Y=1|M=m)} \\
&= \frac{\sum_{a=0}^{1}\Pr(Y=1\mid A=a,M=m)\Pr(A=a|M=m) - \sum_{a=0}^{1}\Pr(Y^1=1\mid A=a,M=m)\Pr(A=a|M=m)}{\Pr(Y=1|M=m)} \\
&= \frac{\Pr(A=0|M=m)\,(\Pr(Y=1\mid A=0,M=m)-\Pr(Y^1=1\mid A=0,M=m))}{\Pr(Y=1|M=m)} \\
&= \frac{\Pr(A=0)\Pr(M=m|A=0)\,(\Pr(Y=1\mid A=0,M=m)-\Pr(Y^1=1\mid A=0,M=m))}{\Pr(Y=1,M=m)}.
\end{aligned}
$$

Thus, the following equation holds:

$$PF_m \Pr(Y = 1, M = m) = \Pr(A = 0) \Pr(M = m|A = 0) \left(\Pr(Y = 1 \mid A = 0, M = m) - \Pr(Y^1 = 1 \mid A = 0, M = m)\right).$$

By plugging this into the expression for the preventable fraction in the total population, we obtain

$$\begin{aligned} PF_{total} &= \frac{\Pr(A = 0) \sum_m (\Pr(Y = 1 \mid A = 0, M = m) - \Pr(Y^1 = 1 \mid A = 0, M = m)) \Pr(M = m|A = 0)}{\Pr(Y = 1)} \\ &= \frac{\sum_m PF_m \Pr(Y = 1, M = m)}{\Pr(Y = 1)} \\ &= \sum_m PF_m \Pr(M = m| Y = 1). \end{aligned}$$

Note the similarity between the formula above and the second formula in the Theorem 2.2 of the preventable fraction (population) in Table S2 of Suzuki and Yamamoto [1].

Derivation for the attributable fraction

Under the consistency assumption, the attributable fraction in the total population can be expressed in terms of the strata of M as follows:

$$AF_{total} \triangleq \frac{\Pr(Y=1) - \Pr(Y^0=1)}{\Pr(Y=1)}$$

$$= \frac{\sum_{a=0}^{1} \Pr(Y=1 \mid A=a) \Pr(A=a) - \sum_{a=0}^{1} \Pr(Y^0=1 \mid A=a) \Pr(A=a)}{\Pr(Y=1)}$$

$$= \frac{\Pr(A=1) \left(\Pr(Y=1 \mid A=1) - \Pr(Y^0=1 \mid A=1)\right)}{\Pr(Y=1)}$$

$$= \frac{\Pr(A=1) \sum_m \left(\Pr(Y=1 \mid A=1,\ M=m) - \Pr(Y^0=1 \mid A=1,\ M=m)\right) \Pr(M=m|A=1)}{\Pr(Y=1)}.$$

The third equation holds because $\Pr(Y=1 \mid A=0) = \Pr(Y^0=1 \mid A=0)$ holds. Similarly, the m-level attributable fraction is expressed as follows:

$$AF_m \triangleq \frac{\Pr(Y=1|M=m) - \Pr(Y^0=1|M=m)}{\Pr(Y=1|M=m)}$$

$$= \frac{\sum_{a=0}^{1} \Pr(Y=1 \mid A=a, M=m) \Pr(A=a|M=m) - \sum_{a=0}^{1} \Pr(Y^0=1 \mid A=a, M=m) \Pr(A=a|M=m)}{\Pr(Y=1|M=m)}$$

$$= \frac{\Pr(A=1|M=m) \left(\Pr(Y=1 \mid A=1, M=m) - \Pr(Y^0=1 \mid A=1, M=m)\right)}{\Pr(Y=1|M=m)}$$

$$= \frac{\Pr(A=1) \Pr(M=m|A=1) \left(\Pr(Y=1 \mid A=1, M=m) - \Pr(Y^0=1 \mid A=1, M=m)\right)}{\Pr(Y=1, M=m)}.$$

Thus, the following equation holds:

$$AF_m \Pr(Y = 1, M = m) = \Pr(A = 1) \Pr(M = m|A = 1) \left(\Pr(Y = 1 \mid A = 1, M = m) - \Pr(Y^0 = 1 \mid A = 1, M = m)\right).$$

By plugging this into the expression for the attributable fraction in the total population, we obtain

$$\begin{aligned} AF_{total} &= \frac{\Pr(A = 1) \sum_m (\Pr(Y = 1 \mid A = 1, M = m) - \Pr(Y^0 = 1 \mid A = 1, M = m)) \Pr(M = m|A = 1)}{\Pr(Y = 1)} \\ &= \frac{\sum_m AF_m \Pr(Y = 1, M = m)}{\Pr(Y = 1)} \\ &= \sum_m AF_m \Pr(M = m| \, Y = 1). \end{aligned}$$

Note the similarity between the formula above and the second formula in the Theorem 1.2 of the attributable fraction (population) in Table 1 of Suzuki and Yamamoto [1].

Derivation for the preventable proportion

Under the consistency assumption, the preventable proportion in the total population can be expressed in terms of the strata of M as follows:

$$
\begin{aligned}
PP_{total} &\triangleq \frac{\Pr(Y=1)-\Pr(Y=1,Y^1=1)}{\Pr(Y=1)} \\
&= \frac{\sum_{a=0}^{1}\Pr(Y=1\mid A=a)\Pr(A=a)\ -\ \sum_{a=0}^{1}\Pr(Y=1,Y^1=1\mid A=a)\Pr(A=a)}{\Pr(Y=1)} \\
&= \frac{\Pr(A=0)\,(\Pr(Y=1\mid A=0)-\ \Pr(Y=1,Y^1=1\mid A=0))}{\Pr(Y=1)} \\
&= \frac{\Pr(A=0)\sum_m(\Pr(Y=1\mid A=0,\ M=m)-\Pr(Y=1,Y^1=1\mid A=0,\ M=m))\Pr(M=m|A=0)}{\Pr(Y=1)}.
\end{aligned}
$$

The third equation holds because $\Pr(Y=1\mid A=1)=\Pr(Y=1,Y^1=1\mid A=1)$ holds. Similarly, the m-level preventable proportion is expressed as follows:

$$
\begin{aligned}
PP_m &\triangleq \frac{\Pr(Y=1|M=m)-\Pr(Y=1,Y^1=\ 1|M=m)}{\Pr(Y=1|M=m)} \\
&= \frac{\sum_{a=0}^{1}\Pr(Y=1\mid A=a,M=m)\Pr(A=a|M=m)\ -\ \sum_{a=0}^{1}\Pr(Y=1,Y^1=1\mid A=a,M=m)\Pr(A=a|M=m)}{\Pr(Y=1|M=m)} \\
&= \frac{\Pr(A=0|M=m)\,(\Pr(Y=1\mid A=0,M=m)-\Pr(Y=1,Y^1=1\mid A=0,M=m))}{\Pr(Y=1|M=m)} \\
&= \frac{\Pr(A=0)\Pr(M=m|A=0)\,(\Pr(Y=1\mid A=0,M=m)-\Pr(Y=1,Y^1=1\mid A=0,M=m))}{\Pr(Y=1,M=m)}.
\end{aligned}
$$

Thus, the following equation holds:

$$PP_m \Pr(Y=1, M=m) = \Pr(A=0) \Pr(M=m|A=0) \left(\Pr(Y=1 \mid A=0, M=m) - \Pr(Y=1, Y^1=1 \mid A=0, M=m)\right).$$

By plugging this into the expression for the preventable proportion in the total population, we obtain

$$\begin{aligned} PP_{total} &= \frac{\Pr(A=0) \sum_m \left(\Pr(Y=1 \mid A=0, M=m) - \Pr(Y=1, Y^1=1 \mid A=0, M=m)\right) \Pr(M=m|A=0)}{\Pr(Y=1)} \\ &= \frac{\sum_m PP_m \Pr(Y=1, M=m)}{\Pr(Y=1)} \\ &= \sum_m PP_m \Pr(M=m| \, Y=1). \end{aligned}$$

Derivation for the attributable proportion

Under the consistency assumption, the attributable proportion in the total population can be expressed in terms of the strata of M as follows:

$$AP_{total} \triangleq \frac{\Pr(Y=1) - \Pr(Y=1, Y^0=1)}{\Pr(Y=1)}$$
$$= \frac{\sum_{a=0}^{1}\Pr(Y=1 \mid A=a)\Pr(A=a) - \sum_{a=0}^{1}\Pr(Y=1, Y^0=1 \mid A=a)\Pr(A=a)}{\Pr(Y=1)}$$
$$= \frac{\Pr(A=1)\left(\Pr(Y=1 \mid A=1) - \Pr(Y=1, Y^0=1 \mid A=1)\right)}{\Pr(Y=1)}$$
$$= \frac{\Pr(A=1)\sum_m\left(\Pr(Y=1 \mid A=1,\ M=m) - \Pr(Y=1, Y^0=1 \mid A=1,\ M=m)\right)\Pr(M=m|A=1)}{\Pr(Y=1)}.$$

The third equation holds because $\Pr(Y=1 \mid A=0) = \Pr(Y=1, Y^0=1 \mid A=0)$ holds. Similarly, the m-level attributable proportion is expressed as follows:

$$AP_m \triangleq \frac{\Pr(Y=1|M=m) - \Pr(Y=1, Y^0=1|M=m)}{\Pr(Y=1|M=m)}$$
$$= \frac{\sum_{a=0}^{1}\Pr(Y=1 \mid A=a, M=m)\Pr(A=a|M=m) - \sum_{a=0}^{1}\Pr(Y=1, Y^0=1 \mid A=a, M=m)\Pr(A=a|M=m)}{\Pr(Y=1|M=m)}$$
$$= \frac{\Pr(A=1|M=m)\left(\Pr(Y=1 \mid A=1, M=m) - \Pr(Y=1, Y^0=1 \mid A=1, M=m)\right)}{\Pr(Y=1|M=m)}$$
$$= \frac{\Pr(A=1)\Pr(M=m|A=1)\left(\Pr(Y=1 \mid A=1, M=m) - \Pr(Y=1, Y^0=1 \mid A=1, M=m)\right)}{\Pr(Y=1, M=m)}.$$

Thus, the following equation holds:

$$AP_m \Pr(Y=1, M=m) = \Pr(A=1)\Pr(M=m|A=1)\left(\Pr(Y=1 \mid A=1, M=m) - \Pr(Y=1, Y^0=1 \mid A=1, M=m)\right).$$

By plugging this into the expression for the attributable proportion in the total population, we obtain

$$\begin{aligned} AP_{total} &= \frac{\Pr(A=1)\sum_m (\Pr(Y=1 \mid A=1, M=m) - \Pr(Y=1, Y^0=1 \mid A=1, M=m))\Pr(M=m|A=1)}{\Pr(Y=1)} \\ &= \frac{\sum_m AP_m \Pr(Y=1, M=m)}{\Pr(Y=1)} \\ &= \sum_m AP_m \Pr(M=m| \, Y=1). \end{aligned}$$

Reference

1. Suzuki, E. and E. Yamamoto, *Attributable fraction and related measures: Conceptual relations in the counterfactual framework.* Journal of Causal Inference, 2023. **11**. 20210068

eFigure 1. Relationships between attributable and preventable fractions, average causal effects (ACE), target population, and target group of causation. Regarding target populations, we assume that both AF_{total} and PF_{total} as well as the average causal effects under consideration are defined for the entire population; that is, they are not defined via conditioning on other variables, including potential outcomes variables. By target of causation, it is meant the subset of the population for which there must be a non-zero contrast between the (average) observed and the relevant counterfactual outcomes, so that the AF_{total}, or PF_{total}, is non-zero; for the average causal effect, this corresponds to (the potential subset of) the population included in the contrast of average potential outcomes.

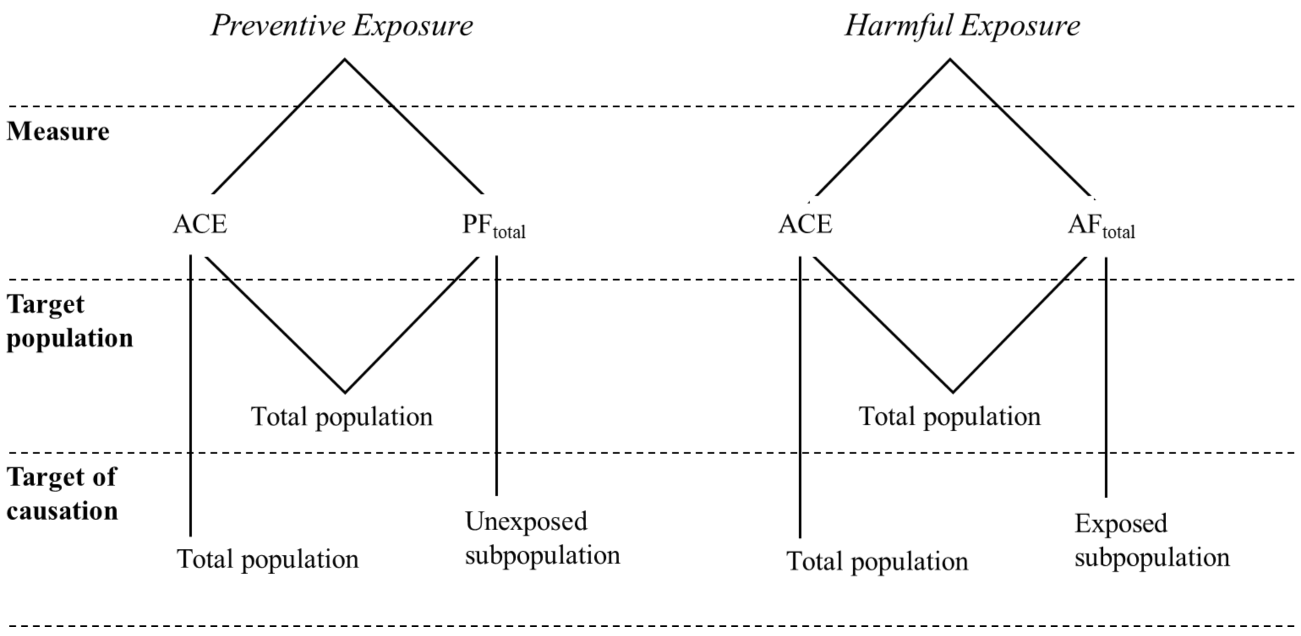

Supplement: Supplementary file 1 [file je-36-168-s001.pdf]
